# Community-Level Factors Associated With Geographic Access to Food Retailers Offering Nutrition Incentives in Chicago, Illinois

**DOI:** 10.5888/pcd19.210211

**Published:** 2022-02-10

**Authors:** Chelsea R. Singleton, Fikriyah Winata, Alexandra M. Roehll, Isa Adamu, Gabriella M. McLoughlin

**Affiliations:** 1 Department of Social, Behavioral, and Population Sciences, Tulane School of Public Health and Tropical Medicine, New Orleans, Louisiana; 2 Department of Geography and Geographic Information Science, University of Illinois at Urbana–Champaign, Urbana, Illinois; 3 Department of Kinesiology and Community Health, University of Illinois at Urbana–Champaign, Champaign, Illinois; 4 Department of Kinesiology, Temple University College of Public Health, Philadelphia, Pennsylvania; 5 Implementation Science Center for Cancer Control and Prevention Research Center, Brown School, Washington University in St. Louis, St. Louis, Missouri

## Abstract

**Introduction:**

Nutrition incentive programs provide low-income populations with a monetary resource to make healthy foods affordable and accessible. This study aimed to use geospatial analysis to evaluate availability of the Link Match nutrition incentive program in Chicago, Illinois, to determine whether underresourced communities have access.

**Methods:**

We obtained 2018 spatial data on census tract–level sociodemographic characteristics in Chicago. Fifty-seven retailers (eg, farmers markets, food cooperatives) offered Link Match across the city’s 801 census tracts. We examined ordinary least squares and spatial lag regression models to identify census tract–level variables associated with distance (in miles) from the nearest Link Match retailer. Variables of interest included percentage of non-Hispanic Black residents, percentage of Hispanic residents, median household income, violent crime rate, per capita grocery store availability, and walkability.

**Results:**

Most Link Match retailers were located on Chicago’s South and West sides. Ordinary least squares regression models indicated that low-income census tracts were on average closer to a Link Match retailer than higher-income tracts were (*P* < .001). Tracts in the highest quartile of violent crime were also significantly closer to a Link Match retailer than tracts in the lowest quartile (*P* < .001). After accounting for spatial dependency of census tracts, only violent crime rate was significantly associated with distance to nearest Link Match retailer.

**Conclusion:**

Link Match retailers in Chicago appear to be in underresourced communities. However, these areas have high violent crime rates, which may negatively influence program use. Additional research is needed on how social and environmental factors influence availability and use of nutrition incentive programs.

**Summary**

**What is already known on this topic?**

Nutrition incentive programs make healthy foods more affordable and accessible for low-income populations. In Chicago, Illinois, several retailers, including farmers markets, farm stands, and food cooperatives, offer nutrition incentives.

**What is added by this report?**

Retailers offering nutrition incentives in Chicago are geographically closer to low-income communities and areas with high violent crime rates compared with higher-income communities and areas with low violent crime rates.

**What are the implications for public health practice?**

Nutrition incentive programs should consider community-level social and environmental factors that may hinder program access among target populations.

## Introduction

Socioeconomic disparities in diet-related diseases (eg, obesity, type 2 diabetes) is a major public health concern (1,2). Low-income populations often face structural barriers to maintaining a healthy diet: limited access to healthy retailers (eg, grocery stores), greater access to unhealthy retailers (eg, convenience stores), and higher costs of healthy items, particularly fresh produce (3–5). Nutrition incentive programs address these barriers by making healthy foods more affordable and accessible (6). Many incentive programs aim to help participants in federal nutrition assistance programs, such as the Supplemental Nutrition Assistance Program (SNAP) (6). Overall, research indicates that incentives positively affect the diets and shopping behaviors of low-income populations (7–9).

In Chicago, Illinois, access to healthy food in low-income communities, specifically majority Black low-income communities, has been low for decades (10,11). Link Match, the largest nutrition incentive program in Illinois, aims to close this gap by offering SNAP participants a one-to-one dollar match (up to $25) if they redeem their benefits at a participating retailer (12,13). This match incentive allows participants to take home double the amount of SNAP-eligible staple food items, which include fruits, vegetables, bread, meat, and dairy. More than 50 retailers in Chicago offer the program; however, local public health professionals and community leaders have limited understanding of the program’s spatial reach. To address this gap in knowledge, we aimed to use geospatial analysis to identify sociodemographic and environmental factors correlated with program access. By doing so, we can determine whether Link Match in Chicago is accessible to populations who are nutritionally vulnerable. Overall, we believe this research will be of interest to organizations that operate nutrition incentive programs or work in the space of nutrition equity.

## Methods

We used ArcGIS version 10.8.1 software (Esri) to examine census tract–level factors associated with distance to the nearest Link Match retailer in Chicago. Census tract was the unit of analysis. We calculated the point-to-point distance (in miles) to the nearest Link Match retailer for all Chicago census tracts (N = 801) using the centroid of the tract as the reference point. We obtained a list of Link Match retailers that operated in summer 2020 from Experimental Station, the nonprofit organization that runs Link Match (12). We mapped 57 retailer locations in Chicago where residents could access the program: 29 farmers markets (50.9%), 9 mobile market stops (15.8%), 8 food cooperatives (14.0%), 5 farm stands (8.8%), and 6 “other” markets (10.5%). “Other” markets were those that labeled themselves a “health market” or “community food market.” The institutional review board at the University of Illinois at Urbana–Champaign deemed this exempt research.

### Variables

We gathered census tract–level data on sociodemographic and environmental variables from various sources (14–17). We obtained 2018 data on the following sociodemographic variables from the US Census: percentage of the census tract population that was non-Hispanic Black, percentage of the census tract population that was Hispanic, median annual household income, and population size (14). These data represent 5-year American Community Survey estimates (14). We categorized census tract–level median household income into 2 categories: lower income and higher income. Lower-income census tracts were those that had a median household income lower than the city’s median in 2018 ($55,295). Higher-income tracts were defined as those with a median income of $55,295 or more. We used the population size estimate to calculate population density for each census tract, defined as the number of people per square mile. Our environmental variables of interest were violent crime rate, per capita grocery store availability, and walkability. Chicago’s Citizen Law Enforcement and Reporting (CLEAR) system collects data on police-reported crime events throughout the year (15). We defined violent crime rate as the number of police-reported homicides, armed robberies, and aggravated assault incidents per 1,000 residents in 2018. Because of the large volume of crimes reported and the skewed distribution across the city, we categorized census tracts into quartiles of violent crime rate. Census tracts in the lowest quartile of violent crime rate was the reference group. Data on grocery store locations were available from the Chicago Data Portal (16). We defined per capita grocery stores as the number of stores per 1,000 residents in 2020. Lastly, we obtained 2017 data on walkability from the US Environmental Protection Agency (17). Walkability was measured by using the National Walkability Index (range, 1–20); a higher score indicates a more walkable area (17).

### Statistical analysis

We calculated descriptive statistics (ie, means and SDs) for all variables of interest. We used GeoDa 1.18 software (https://geodacenter.github.io) to examine unadjusted and adjusted ordinary least squares (OLS) and spatial lag models to identify sociodemographic and environmental factors associated with distance in miles to the nearest Link Match retailer. Each unadjusted model included only 1 variable of interest. The adjusted model included all sociodemographic and environmental variables. Spatial lag models accounted for any spatial dependency that may have existed among census tracts. We assessed significance at the α level of .05.

## Results

**Figure 1:**
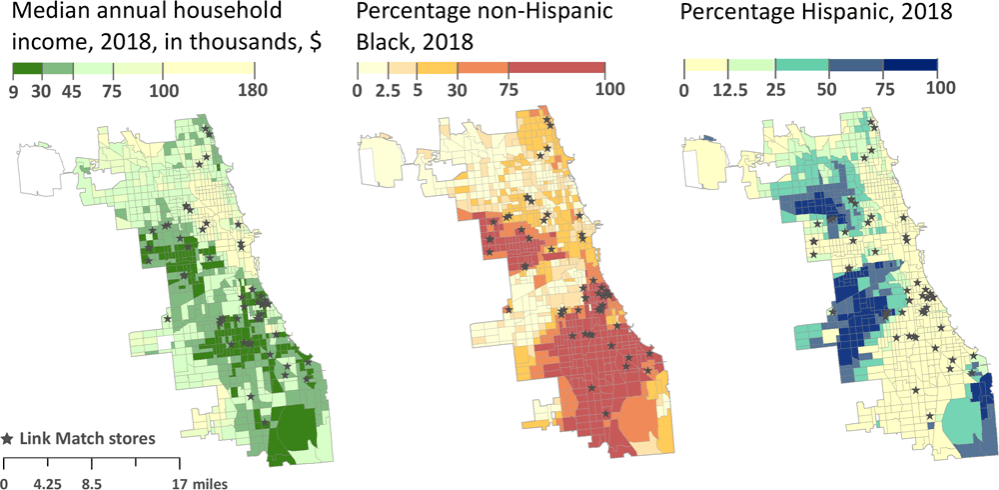
Link Match retailers mapped onto 3 sociodemographic variables: median annual household income in 2018, percentage of the population that was non-Hispanic Black in 2018, and percentage of population that was Hispanic in 2018, by census tract (N = 801), Chicago, Illinois. Map created in ArcGIS software version 10.8.1 (Esri). Data source: US Census Bureau (14).

Many Link Match retailers were located on the city’s South and West sides. Of the 57 retailers, 39 (68.4%) were in majority non-Hispanic Black census tracts and 5 (8.8%) were in majority Hispanic census tracts (Figure 1). Furthermore, 44 (77.2%) were in lower-income census tracts. Across all census tracts, the average distance to the nearest Link Match retailer was 1.4 (SD, 1.3) miles (Table 1).

**Table 1:** Descriptive Characteristics of Census Tracts (N = 801) in Chicago, Illinois

Variable	All Census Tracts, Mean (SD)
**Sociodemographic**	
% Non-Hispanic Black^a^	36.1 (39.8)
% Hispanic^a^	25.8 (28.7)
Median annual household income, $^a^	57,084 (32,387)
Population density, per square mile	20,547 (36,443)
**Environmental**	
Distance to nearest Link Match^b^ retailer, mile	1.4 (1.3)
Violent crime rate^c^	2.74 (3.13)
No. of grocery stores per 1,000 residents^d^	0.10 (0.22)
National Walkability Index^e^	12.5 (2.3)

a Data source: US Census Bureau (14); 2018 estimates.

b Link Match is the largest nutrition incentive program in Illinois; it offers Supplemental Nutrition Assistance Program participants a one-to-one dollar match (up to $25) if they redeem their benefits at a participating retailer (12,13).

c Data source: Chicago Police Department (15). Number of police-reported violent crime events (ie, homicide, armed robbery, aggravated assault) per 1,000 residents in 2018.

d Data source: Chicago Data Portal (16); 2020 data.

e Based on the US Environmental Protection Agency’s National Walkability Index (range, 0–20), with higher scores indicating greater walkability (17); 2017 estimates.

**Figure 2:**
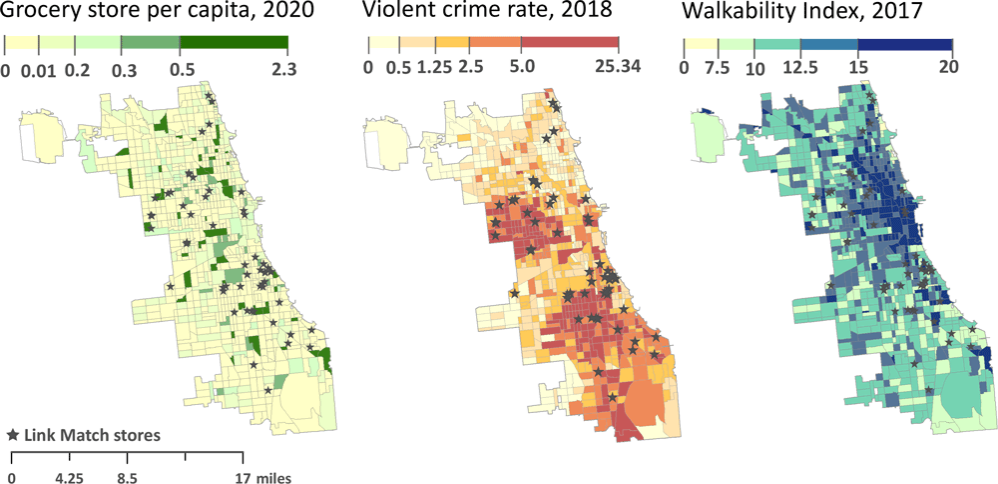
Link Match retailer locations mapped to 3 environmental variables: number of grocery stores per capita in 2020, violent crime rate in 2018, and the National Walkability Index in 2017, by census tract (N = 801), Chicago, Illinois. Violent crime and grocery store location data were obtained from the Chicago Data Portal (16). Violent crime rate was defined as the number of police-reported incidents of homicide, armed robbery, and aggravated assault per 1,000 census tract residents. Per capita grocery stores was defined as the number of grocery stores per 1,000 census tract residents. Data on walkability were obtained from the US Environmental Protection Agency; the higher the National Walkability Index score (scale, 0–20), the more walkable the census tract. Map created in ArcGIS software version 10.8.1 (Esri).

The average violent crime rate was 2.7 (SD, 3.1) events per 1,000 residents. Census tracts, on average, had less than 1 grocery store per 1,000 tract residents, and the average National Walkability Index score was 12.5 (SD, 2.3) (Figure 2).

Unadjusted OLS models, which did not account for spatial dependency, indicated that all variables of interest were associated with distance, except per capita grocery stores, National Walkability Index score, and population density (Table 2). The negative coefficient estimates suggested that census tracts with higher percentages of non-Hispanic Black residents (*P* < .001), a lower income (*P* < .001), and higher violent crime rates (*P* < .001 for all quartiles) were closer in distance to Link Match retailers. However, in the adjusted OLS model, we found lower income, violent crime rate (*P* < .001 for all quartiles), and National Walkability Index (*P* < .001) to be significant. On average, lower-income census tracts were 0.37 miles closer than higher-income census tracts to a Link Match retailer. On average, census tracts in the highest quartile of violent crime rate were 0.96 miles closer to a Link Match retailer than census tracts in the lowest quartile. Furthermore, every 1-unit increase in the National Walkability Index score was associated with a 0.08-mile decline in distance to a retailer.

**Table 2:** Regression Models Examining Sociodemographic and Environmental Variables Associated With Distance to Nearest Link Match Retailer in Census Tracts (N = 801) in Chicago, Illinois^a^

Variable	Ordinary Least Squares Crude Models	Ordinary Least Squares Adjusted Model^b^	Spatial Lag Crude Models	Spatial Lag Adjusted Model^b^
% Non-Hispanic Black^c^	−0.01 (0.001) [<.001]	−0.003 (0.002) [.08]	−0.0005 (0.0002) [.02]	0.0002 (0.0004) [.95]
% Hispanic^c^	0.004 (0.002) [.009]	0.001 (0.002) [.59]	0.0002 (0.0003) [.38]	0.0002 (0.0004) [.65]
Median annual household income, $^c^				
<55,295	−0.77 (0.09) [<.001]	−0.37 (0.11) [<.001]	−0.03 (0.02) [.06]	−0.01 (0.02) [.59]
≥55,295	1 [Reference]	1 [Reference]	1 [Reference]	1 [Reference]
Violent crime rate per 1,000 residents^d^				
Quartile 1 (0–0.61)	1 [Reference]	1 [Reference]	1 [Reference]	1 [Reference]
Quartile 2 (0.62–1.52)	−0.84 (0.11) [<.001]	−0.74 (0.12) [<.001]	−0.04 (0.02) [.08]	−0.04 (0.02) [.09]
Quartile 3(1.53–3.74)	−1.22 (0.11) [<.001]	−0.98 (0.13) [<.001]	−0.06 (0.02) [.006]	−0.05 (0.02) [.03]
Quartile 4 (3.75–25.34)	−1.41 (0.11) [<.001]	−0.96 (0.16) [<.001]	−0.08 (0.02) [<.001]	−0.07 (0.03) [.03]
No. of grocery stores per 1,000 residents^e^	−0.19 (0.20) [.36]	0.05 (0.18) [.78]	−0.03 (0.03) [.33]	−0.02 (0.03) [.61]
National Walkability Index^f^	−0.03 (0.02) [.09]	−0.08 (0.02) [<.001]	−0.003 (0.003) [.31]	−0.005 (0.003) [.21]
Census-tract population density per square mile^c^	0.0000008 (0.000001) [.42]	−0.000001 (0.000001) [.17]	0.0000003 (0.0000002) [.08]	0.0000002 (0.0000002) [.26]
Spatial lag (*W*)^g^	—	—	—	0.99 (0.003) [<.001]

Abbreviation: —, does not apply.

a Link Match is the largest nutrition incentive program in Illinois; it offers Supplemental Nutrition Assistance Program participants a one-to-one dollar match (up to $25) if they redeem their benefits at a participating retailer (12,13). All values are β (SE) [*P* value].

b Fully adjusted models include all variables.

c Data source: US Census Bureau (14); 2018 estimates.

d Data source: Chicago Police Department (15). Number of police-reported violent crime events (ie, homicide, armed robbery, aggravated assault) per 1,000 residents in 2018.

e Data source: Chicago Data Portal (16); 2020 data.

f Based on the US Environmental Protection Agency’s National Walkability Index (range, 0–20), with higher scores indicating greater walkability (17); 2017 estimates.

g The spatial regression model term that accounts for spatial correlation among census tracts in Chicago.

Unadjusted spatial lag models indicated that only the variables for percentage of non-Hispanic Black residents (*P* = .02) and the 2 highest quartiles of violent crime rate (both *P* < .01) were significantly associated with distance to nearest Link Match retailer. The adjusted spatial lag model indicated that only the violent crime rate retained significance.

## Discussion

To gain a better understanding of which areas of Chicago have access to food retailers offering the Link Match incentive program in the summer months, we performed geospatial analyses on census tract–level data collected from several sources. Our findings showed that lower median income and a lower violent crime rate were negatively associated with distance to a Link Match retailer. This finding suggests that Chicago census tracts with more low-income residents and higher violent crime rates are closer in distance to a Link Match retailer. Most Link Match retailers are located on the city’s South and West sides, which have large populations of low-income non-Hispanic Black residents. This proximity to Link Match retailers is ideal given Chicago’s gaps in healthy food access, which have historically affected low-income communities and people of color (10,11). Nevertheless, it is important to keep in mind that many Link Match retailers are farmers markets and farm stands that operate in summer only. In Illinois, market season typically runs from May through August, so many Link Match retailers are not available during the off-season. Distance to a Link Match retailer may increase for some or most census tracts in Chicago outside market season. Seasonality of direct-to-consumer retailers, such as farmers markets, has been cited as a barrier to regularly accessing nutrition incentives (18,19). Thus, location and seasonality may be 2 key barriers to accessing Link Match in Chicago.

Crime may also be a barrier to program access, but scientific evidence on this topic is limited. Chicago census tracts with a higher violent crime rate were closer to Link Match retailers. Most Link Match retailers operate outdoors (eg, farmers markets, farm stands) in summer when crime rates are highest in Chicago (20). Public health research has found that community members do not readily use outdoor community spaces, such as public parks, immediately after violent crime events (21). Furthermore, sociological research has reported that fear of crime, violence, and discrimination can limit an individual’s participation in community-organized events, such as farmers markets (22). If a connection exists between crime and use of direct-to-consumer retailers, relevant community leaders and interested parties should position public safety as a strategy to increasing use of nutrition incentive programs. Nevertheless, information in the literature is limited on the role of crime rates in accessing retailers that offer nutrition incentives. To expand the field’s understanding of socioenvironmental factors that influence nutrition incentive program access, additional research is needed.

Our research has limitations. Although our analysis featured geospatial data collected by various reliable sources (US Census Bureau, US Environmental Protection Agency, and the City of Chicago), the study was an ecological assessment conducted at the census tract level in Chicago. We cannot draw conclusions about community-level factors associated with individual-level proximity to Link Match retailers among Chicago residents. We used the center point of the census tract as the reference point when calculating distance to the nearest Link Match retailer for every tract. Given the shape of each census tract, the street connectivity of roadways, and the spatial distribution of tract residents, the calculated distance may not be a precise representation of the actual distance (in miles) it takes a resident to travel to a Link Match retailer. For ease of interpretation, we categorized the continuous variables for median household income and violent crime rate. However, sensitivity analyses indicated that findings from models using all continuous variables were similar to the findings of the models presented here. Finally, because recent data for some variables were not available (eg, National Walkability Index), the years represented in the data are not consistent across variables and sources. However, we do not believe community amenities or the built environment changed sufficiently in Chicago from 2017 to 2020 to have affected our study findings.

Future research on factors influencing access to nutrition incentives and other food assistance programs should devote more attention to studying barriers, such as location, seasonality of retailers, violent crime, and other relevant social and contextual factors. Despite efforts to make healthy food more affordable and accessible, individual-level data suggest that structural barriers may be preventing populations that are nutritionally vulnerable in Chicago from using services such as Link Match (23). Conducting studies that incorporate individual-level data on use of nutrition incentives and community-level data on program accessibility will be particularly important.

In summary, our findings underscore the importance of considering community-level sociodemographic and environmental factors and their influence on access to nutrition incentive programs. When developing strategies and programming that address nutritional inequities, experts and community organizations should ensure equitable access to healthy foods and reduce deterrents (ie, social exclusion, discrimination, threats to public safety) to healthy behaviors (24). Historically underresourced communities, such as low-income communities and communities of color, are disproportionately affected by structural barriers, which increases residents’ risk of food insecurity and obesity (25–27). Thus, an explicit emphasis needs to be placed on addressing social determinants of health, such as food accessibility and affordability, as means to improve the diets and health of these populations.
